# Influenza vaccine uptake among children and older adults in China: a secondary analysis of a quasi-experimental study

**DOI:** 10.1186/s12879-023-08145-8

**Published:** 2023-04-13

**Authors:** Yumeng Du, Chenqi Jin, Mark Jit, Tracey Chantler, Leesa Lin, Heidi J. Larson, Jing Li, Wenfeng Gong, Fan Yang, Nina Ren, Weibin Cheng, Yi Zhou, Weiming Tang, Joseph D. Tucker, Dan Wu

**Affiliations:** 1 Institute for Healthcare Artificial Intelligence Application, Guangdong Second Provincial General Hospital, Guangzhou, China; 2 Department of Clinical Research, Faculty of Infectious and Tropical Diseases, London School of Hygiene and Tropical Medicine, London, UK; 3 University of North Carolina at Chapel Hill Project-China, Guangzhou, China; 4 Dermatology Hospital of South Medical University, Guangzhou, China; 5 Faculty of Epidemiology and Population Health, London School of Hygiene and Tropical Medicine, London, UK; 6 Laboratory of Data Discovery for Health (D24H), Hong Kong Science Park, New Territories, Hong Kong China; 7 Faculty of Public Health and Policy, London School of Hygiene and Tropical Medicine, London, UK; 8 Department of Occupational and Environmental Health, West China School of Public Health, Sichuan University, Chengdu, China; 9 China Country Office, Bill & Melinda Gates Foundation, Beijing, China; 10 Institute of Population Research, Peking University, Beijing, China; 11 Zhuhai Center for Disease Control and Prevention, Zhuhai, China; 12 Institute for Global Health and Infectious Diseases, School of Medicine, University of North Carolina at Chapel Hill, Chapel Hill, NC USA; 13 Department of Clinical Research, London School of Hygiene & Tropical Medicine, Room 360, Keppel St, London, WC1E 7HT UK

**Keywords:** Influenza vaccines, Children, Older adults, China

## Abstract

**Background:**

Influenza vaccination is the key to prevent influenza-related disease, especially among high-risk populations. However, influenza vaccine uptake in China is low. This secondary analysis of a quasi-experimental trial aimed to understand factors associated with influenza vaccine uptake among children and older people stratified by funding context.

**Methods:**

A total of 225 children (aged 0.5-8 years) and 225 older people (aged 60 years or above) were recruited from three clinics (rural, suburban and urban) in Guangdong Province. Participants were allocated into two groups based on funding contexts: a self-paid group (N = 150, 75 children and 75 older adults) in which participants paid full price for their vaccination; and a subsidized group (N = 300, 150 children and 150 older adults) in which varying levels of financial support was provided. Univariate and multivariable logistic regressions were conducted stratified by funding contexts.

**Results:**

Overall, 75.0% (225/300) of participants in the subsidized group and 36.7% (55/150) in the self-paid group got vaccinated. Older adults had lower vaccination rates than children in both funding groups, while both age groups showed much higher uptake in the subsidized group than in the self-paid group (aOR = 5.96, 95% CI: 3.77–9.42, p = 0.001). In the self-paid group, having prior influenza vaccination history of children (aOR:2.61, 95%CI: 1.06–6.42) or older people (aOR:4.76, 95%CI: 1.08–20.90) was associated with increased influenza vaccine uptake compared to those who had no prior vaccination experiences in the family. While in the subsidized group, participants who got married or lived with partners (aOR = 0.32, 0.10–0.98) had lower vaccination uptake than single ones. Trust in providers’ advice (aOR = 4.95, 95%CI:1.99, 12.43), perceived effectiveness of the vaccine (aOR: 12.18, 95%CI: 5.21–28.50), and experienced influenza-like illnesses in the family in the past year (aOR = 46.52, 4.10, 533.78) were associated with higher vaccine uptake.

**Conclusions:**

Older people had suboptimal vaccine uptake compared to children in both contexts and need more attention to enhance influenza vaccination. Tailoring interventions to different vaccine funding contexts may help improve influenza vaccination: In self-paid context, motivating people to accept their first ever influenza vaccination may be a promising strategy. In subsidized context, improving public confidence in vaccine effectiveness and providers’ advice would be useful.

**Supplementary Information:**

The online version contains supplementary material available at 10.1186/s12879-023-08145-8.

## Introduction

Influenza is a respiratory infectious disease causing 300,000 to 500,000 deaths worldwide each year [1]. In China, 88,1000 annual influenza-associated excess deaths were estimated to have occurred on average during 2010–2015 [2]. Older adults and children are both at higher risk of influenza infection and death [3]. Chinese older adults, defined as ≥ 60 years old in the Chinese setting, are 26 times more likely to die from influenza-related illnesses compared to younger adult [2], and Chinese children aged < 18 years old are estimated to have twice the risk of getting influenza as adults [4].

Influenza vaccination is the most effective way to prevent infection and reduce influenza-related disease burden [5]. Systematic reviews concluded that influenza vaccine can prevent nearly two thirds of influenza cases among all adults [6, 7], and more than half of cases among children aged 5–59 months and older adults in China [8, 9]. However, influenza vaccination uptake in China is low. The Chinese Center for Disease Control and Prevention has recommended annual influenza vaccination to high-risk subgroups including older people and children since 2014 [5], but only 3.8% of older people [10] and less than one-fifth of children received an influenza vaccine in the past year [11].

Vaccination-associated user fees could be a barrier and public funding to lower costs can increase influenza vaccination uptake [12, 13]. Public funding policies for influenza vaccine vary by region in mainland China [10]. Economically developed places provide partially (e.g., Huaiyin in Jiangsu province) or fully (e.g., Beijing and Karamay) subsidized vaccines for children and older people via insurance schemes or local government finance [14]. People in most places, however, have to pay the standard market price (USD 12–23) to receive an influenza vaccine [14]. Furthermore, in places where free vaccines are available, the vaccination rates are still sub-optimal compared to many developed countries [14]. During 2021–2022 season, Influenza vaccination uptake among older adults in China’s free vaccine regions (32.9%) [15] was much lower than those in the US (73.9%) [16] and the UK (82.3%) [17]. There is a need to understand factors influencing influenza vaccination uptake other than cost to inform the vaccination scale-up under different Chinese financing context.

Factors influencing vaccine uptake are multi-faceted. Previous systematic reviews identified sociodemographic, knowledge, attitudes (e.g., self-perceived low risks of infection), experiential and contextual factors as barriers and facilitators of influenza vaccine uptake [18–24]. Most previous studies have focused on healthcare workers in high-income countries [18] and on older people [21–24]. Evidence on high-risk populations of influenza, especially from low and middle-income countries (LMICs) [25, 26], is limited. Moreover, most studies of influenza vaccine uptake in China have been limited to urban areas and were conducted before the COVID-19 pandemic [10, 15, 27–29]. Factors influencing vaccine uptake stratified by funding contexts are underexplored.

This is a secondary data analysis of a parent quasi-experimental study (trial registration: ChiCTR2000040048) in which the effectiveness of a pay-it-forward intervention in improving influenza uptake in children and older people was assessed against a standard-of-care arm. Individuals in the pay-it-forward arm received free vaccine pre-paid by others in the community and offered the individual an opportunity to donate voluntarily at any amount to support subsequent people to receive the same service [30]. An additional free arm was included in the parent study primarily for costs comparison. These two free arms were treated in this secondary data analysis to reflect the fully subsidized context and combined with the pay-it-forward arm as subsidized vaccination. This secondary analysis aims to examine the associated factors of influenza vaccine uptake among children and older people in different funding contexts (self-paid vs. subsidized vaccination)in the Chinese setting. Our study can help inform the development of tailored influenza vaccination programs that are sustainable in the contexts of immunization program funding in China.

## Methods

### Study settings and participants

The parent intervention study took place between September 2020 to February 2021 in Guangdong Province of Southern China where influenza is prevalent throughout the year [31]. The study design has been described in detail in the publication of the parent study [30]. Three clinics with relatively stable availability of influenza vaccines were chosen as study sites. These included one vaccination clinic in rural Yangshan county, one community healthcare center located in the suburban Zengcheng district, and one in the downtown area of urban Guangzhou city. At all three study sites, no public funding is available and people self-pay for influenza vaccine services.

Children and older people were included as our study populations and combined for analysis. There are three reasons for this:1) both children and older adults are at-risk populations of influenza infections and priority groups of vaccination in China [5]; 2) influenza vaccination rate of both children and older adults in China are very low [10, 11], and both of these two groups may benefit from our study findings; 3) a comparative perspective on the vaccination status of children and the elderly can be achieved, by adding independent variable of participant type from a statistical point of view. The participant eligibility includes:1) children aged 6 months to 8 years; 2) older adults aged 60 years old or above; 3) none of them have acute moderate or severe illnesses, and with no prior history of severe allergies. We obtained verbal consent from older adults and from children’s caregivers who consented on behalf of their children. Consent statements were recorded online. Research staff assisted participants who had difficulty in reading or using mobile devices for the online survey.

### Parent study design and data collection

**Fig. 1:**
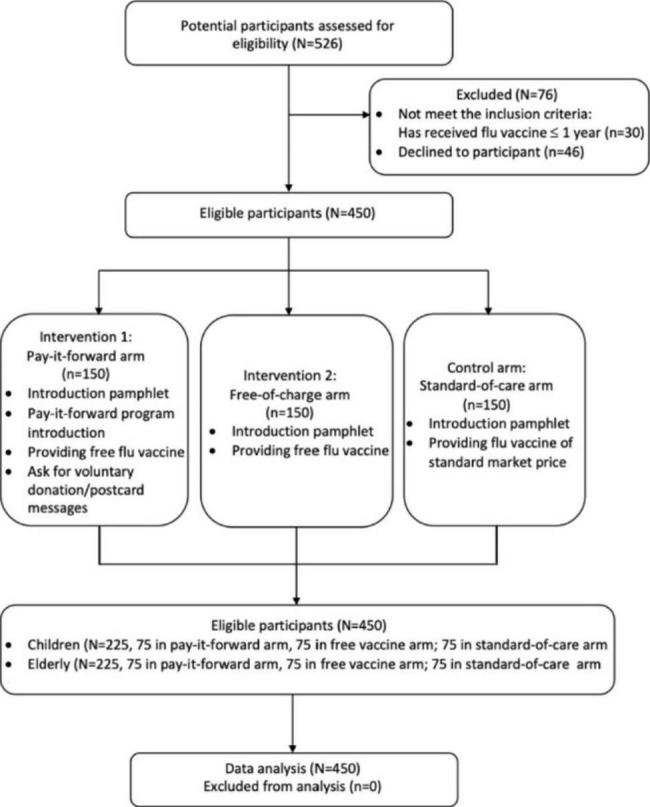
Quasi-experimental study flow chart of the three arms in each site

The intervention study used a quasi-experimental design and included three arms: a standard-of-care arm (i.e. participants had to pay the standard market price for their vaccine), a free-of-charge arm (i.e., participants were provided free influenza vaccine and fees were covered by research costs) and a pay-it-forward arm (i.e., participants received a free influenza vaccination from a local group, and then were asked if they would like to voluntarily donate any amount of money or write postcards to support vaccination for subsequent individuals). Participants in all three arms received introductory pamphlet about influenza and vaccination. The intervention study aimed to compare the effectiveness between pay-it-forward and standard-of-care in influenza vaccine uptake among children and older people, with the free vaccination arm not being powered to test the differences and serving the purpose for costs comparison [30]. The pay-it-forward model is depicted in Appendix Figure A1. A flow chart of the parent quasi-experimental study can be found in Fig. 1. Detailed treatments of participants in the three original arms are shown in Appendix A2.

A non-random approach was adopted for participant recruitment in this trial. Participants recruited were chronologically allocated into the specified study arms. Detailed timeline of participant recruitment in each study arms at each site can be found in the published work of the parent study [30]. Each study site recruited all study groups. At each site, participants were firstly recruited into the standard-of-care arm, followed by the pay-it-forward arm, and finally the free-service arm. Eventually, a total sample of 450 participants were recruited, with 150 participants (75 children and 75 older adults) in each arm. This sample size was estimated based on a pilot study at the rural study site from December 2019 to April 29, 2020 (Appendix A3).

Participation in the study was voluntary and anonymous. All participants were asked to complete a self-reported questionnaire (Appendix B1) after being introduced the intervention. Administrative data recorded by the project staff included the number of eligible people invited, number of individuals who participated and completed a survey, and number of participants who received a vaccination. Data source for secondary analysis consisted of independent variable information from self-reported questionnaire and outcome variable information from administrative data.

### Regrouping study arms for the secondary data analysis

The three study arms were categorized into two groups to reflect varying funding contexts in the Chinese setting: self-paid group (standard-of-care arm, N = 150) and subsidized arm (merged pay-it-forward and free-of-charge arms, N = 300). We had practical and statistical considerations for combining pay-it-forward arm with free-of-charge arm.

First, a combined subsidized group consisting of a free arm (vaccine costs were fully covered) and a voluntary pay-it-forward arm (vaccination fees could be considered partially subsidized) could give a simplified practical understanding of the complex vaccine funding policy in China. It also allows us to identify associated factors of influenza vaccine uptake in a context where some level of public financial support is available, in comparison to a context where people self-pay for vaccination.

Second, the donations in the pay-it-forward arm were made on a complete voluntary basis and did not conflict with the subsidized strategy. Free vaccine was at first given to people in the pay-it-forward arm, they were then being asked whether they would like to donate any amount of money. Additionally, results of analysis from the parent study support the homogeneity of pay-it-forward and free-of-charge arm from an economic point of view. The costs per person vaccinated in the pay-it-forward arm (USD 40.33) were close to those in the free vaccine arm (USD 40.92) [30].

Third, statistically, the sample size calculation was not powered to test differences between free-of-charge and the other two arms in the parent study. The free-of-charge arm only served a purpose for costs evaluation [30]. Additionally, sub-analysis by study arms suggested homogeneity in backgrounds of participants between the pay-it-forward arm and the free-of-charge vaccine arm (Appendix Table C3). Univariable regression analysis of grouped data from pay-it-forward and free-of charge arms showed similar patterns of association between vaccination uptake and explanatory variables, and no significant difference in crude odds ratio between the two arms was found in Mantel-Haenszel analysis (Appendix Table C4). A pooled sample could also provide a higher precision estimate of variance and greater power than those of individual samples [32].

### Variable measurements

#### Guiding conceptual framework

**Fig. 2:**
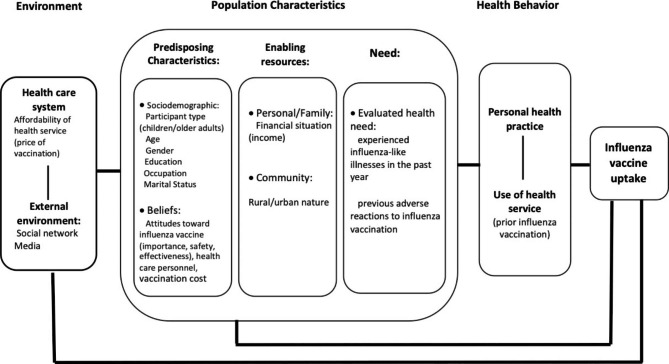
Adapted Anderson behavior Model of potential factors associated with of influenza vaccine uptake

Anderson’s Behavioral Model has been used extensively to understand health service utilization in different health care settings [33]. We adapted the most recent version of Anderson’s Behavioral Model [34] to guide the selection of independent variables and the interpretation of our results (Fig. 2). The adapted model included three major categories of factors that may be associated with influenza vaccination uptake. These categories were population characteristics, behavioral, and environmental factors [34].

Figure 2 depicts potential associated factors within these three categories. Population characteristics included three sub-categories: predisposing characteristics (including individual sociodemographic factors and beliefs), enabling resources and need factors. Individual’s age, sex, participant type (i.e., a child caregiver or an older adult), marital status, education and occupation were included in sociodemographic factors. Belief factors were measured by participants’ attitudes towards influenza vaccine (i.e., perceived importance, safety, and effectiveness of the vaccination), trust in healthcare providers’ advice, and attitudes towards vaccination cost. Sub-category of enabling resources included financial situation (i.e., income) and other community contexts (i.e., rural versus urban settings). In the need sub-category, experienced influenza-like illnesses and adverse reaction to influenza vaccination among family members in the past year as measurements of participants’ self-evaluated health needs. Behavioral category focused on assessing family members’ prior receipt of influenza vaccination. Environmental category focused on levels of affordability of influenza vaccination (i.e., self-paid versus subsidized vaccination as a proxy for health care system influence), exposure to negative news about influenza vaccine (as a proxy for media exposure) and having friends or relatives who were opposed to influenza vaccination (as a proxy for social network influence).

### Statistical analysis

Descriptive analysis was performed to generate frequency distributions of influenza vaccine uptake among children and older people. Differences in sociodemographic characteristics between intervention groups were tested using Pearson’s χ2 test.

We conducted univariate and multivariable logistic regressions for participants in the self-paid and subsidized group respectively, to examine factors influencing Influenza vaccination uptake in different funding context. The binary outcome was ascertained by administrative data and defined as receiving an influenza vaccine or not. All independent variables selected based on the adapted Anderson’s Behavioral Model. Age, sex, and intervention status (i.e., the original pay-it-forward or free-of-charge interventions within the subsidized group) were determined as prior confounders. After the adjustment for prior confounders, each explanatory variable with a significance level of p < 0.2 had a chance to enter the multivariable logistic regression models. Multivariable regression was developed based on the backward stepwise strategy. To avoid sparse data according to approximately ten events per variable [35], a backwards elimination approach based on the Likelihood Ratio test was used. Multicollinearity between explanatory variables was tested by Variance Inflation Factor (VIF) [36]. The age (continuous) variable, with a VIF value of > 5 suggesting multicollinearity among age and other explanatory variable, was dropped from the multivariable models (Appendix Table C5, C6). All analyses were performed using Stata Version 16. A p value of < 0.05 was considered statistically significant.

### Ethical approval

Ethical approval for the parent quasi-experimental study was obtained from the institutional review boards at the London School of Hygiene & Tropical Medicine (Ref: 19100) and the Zhuhai Center for Disease Control (approval number 2020011). This secondary study was approved by the Research Ethics Committee of the London School of Hygiene and Tropical Medicine (Ref: 25292).

## Results

### Characteristics of the participants

Characteristics of the participants by study groups are presented in Table 1. Types of participants (a child caregiver or an older person) were evenly distributed across each study group in line with the age-stratified participant recruitment approach. Participants’ mean age was 52 years old (SD = 17.9), with 53 (SD = 18.9) years in the self-paid group and 52 (SD = 17.3) years in the subsidized group. Most participants (73.3%, 330/450) were women. Missing values of explanatory variables are presented in Appendix Table C2. There were no missing values for the outcome variable (i.e., vaccine uptake).

**Table 1:** Characteristics of participants by study groups in Guangdong Province, China, 2020–2021 (N = 450)

Sociodemographic factors	Total (N = 450) (n,%)	Study groups		*P*-value*
		Self-paid group (N = 150) (n, %)	Subsidized group (N = 300) (n,%)	
**Participant type**				
Children’s caregiver	225 (50.0)	75 (50.0)	150 (50.0)	1.000
Older people	225 (50.0)	75 (50.0)	150 (50.0)	
**Age, mean (SD)**	52 (17.9)	53 (18.9)	52 (17.3)	0.401
Sex				
Male	120 (26.7)	37 (24.7)	83 (27.7)	0.498
Female	330 (73.3)	113 (75.3)	217 (72.3)	
**Completed highest education level**				
Primary school	104 (23.1)	41 (27.3)	63 (21.0)	0.042
Middle school	214 (47.6)	76 (50.7)	138 (46.0)	
Undergraduate or above	132 (29.3)	33 (22.0)	132 (29.3)	
**Occupation**				
Unemployed/retired	234 (52.0)	73 (48.7)	234 (52.0)	0.587
Farmer†	54 (12.0)	20 (13.3)	54 (12.0)	
Employed	162 (36.0)	57 (38.0)	99 (33.0)	
**Marital status**				
Single‡	61 (13.6)	24 (16.0)	37 (12.3)	0.284
Married or living with a partner	389 (86.4)	126 (84.0)	263 (87.7)	

*P-value from chi-square test for comparison of proportion and t test for comparison of mean age

† Farmer refers to people engaged in agriculture, raising living organisms for food or raw materials by leasing land from the government in China, which is different from conventional employed occupations

‡ Single refers to participants who were unmarried, divorced, separated or widowed

### Influenza vaccine uptake

The overall influenza vaccine uptake was 62.2% (280/450), with 36.7% (55/150) of participants in the self-paid group and 75.0% (225/300) in the subsidized group. Vaccine uptake among children and older people are shown in Appendix Figure C1. For both participant types, vaccination uptake in the subsidized group is higher than that in the self-paid group (86.7% vs. 53.3% among children; 63.3% vs. 20.0% among older people). Overall, influenza vaccination rate in subsidized group is nearly 6 times (aOR = 5.96, 95%CI: 3.77–9.42, p = 0.001) of that in self-paid group according to the multivariable logistic analysis (Table 2)

**Table 2:** Multivariable logistic regression to compare influenza vaccine uptake rates between the self-paid and subsidized groups (N = 450)

Different funding group	Crude OR (95% CI)	P-value*	Adjusted OR (95% CI) †	P value‡
Self-paid group (n = 150)	Ref.	< 0.001	Ref.	0.001
Subsidized group (n = 300)	5.18 (3.40, 7.91)		5.96 (3.77, 9.42)	

*P-value for univariate regression analysis

† Adjusted odds ratio: odds ratio adjusted for prior confounders including age and sex in the multivariable logistic model

‡ P value for multivariable regression analysis

### Univariate and multivariable regression analyses of the self-paid group

The results of univariable regression analysis of the self-paid group can be found in Table 3. Older adults were found to have lower vaccination uptake compared to children. Participants with higher completed education level, who were employed, married or living with partners were more likely to receive a vaccine. In terms of beliefs, higher vaccine uptake was reported in participants who recognized the safety and effectiveness of the vaccine, and participants who trusted recommendations from healthcare personnel. Those with an annual income of over USD 1,8600, who reported a prior vaccination history among children or among older people in their family had higher uptake

**Table 3:** Univariable analysis and multivariable analysis of associated factors for influenza vaccine uptake in the self-paid group in Guangdong Province, China, 2020–2021 (N = 150)

Explanatory variables	Crude OR (95% CI)	P-value*	Adjusted OR (95% CI) †	P value‡
**Sociodemographic factors**				
*Participant type*§				
Children’s caregiver	Ref.	< 0.001	Ref.	0.001
Older people	0.22 (0.11, 0.45)		0.19 (0.07, 0.52)	
*Age*	0.95 (0.94, 0.97)	< 0.001	-	-
*Sex*§				
Male	Ref.	0.824	Ref.	0.751
Female	0.19 (0.50, 2.37)		1.17 (0.43, 3.22)	
*Completed highest education level*				
Primary school	Ref.	0.031	-	-
Middle school	3.72 (1.46, 9.46)		-	
Undergraduate or above	4.04 (1.40, 11.73)		-	
*Occupation*§				
Unemployed/retired	Ref.	0.021	-	-
Farmer	0.83 (0.27, 2.56)		-	
Employed	2.56 (1.24, 5.30)		-	
*Marital status*				
Single	Ref.	0.005	-	-
Married or living with a partner	4.92 (1.39, 17.35)		-	
**Belief factors**				
*Importance of influenza vaccine*				
Disagree	Ref.	0.796	-	-
Agree	0.89 (0.38, 2.08)		-	
*Safety of influenza vaccine*				
Disagree	Ref.	0.040	-	-
Agree	2.25 (1.04, 4.86)		-	
*Effectiveness of influenza* § *vaccine*				
Disagree	Ref.	< 0.0001	Ref.	0.242
Agree	4.30 (1.92, 9.62)		1.86 (0.66, 5.21)	
*Trust the advice from medical personnel on influenza vaccine*				
No	Ref.	< 0.0001	-	-
Yes	18.25(2.39,139.18)		-	
*View the price of vaccines as barriers*§				
No	Ref.	0.057	Ref.	0.429
Yes	0.43 (0.18, 1.03)		0.63 (0.18, 1.99)	
**Enabling factors**				
*Annul income (USD)*				
< 1860	Ref.	0.045	-	-
1860 ~ 9300	0.90 (0.40, 2.05)		-	
9300 ~ 1,8600	1.73 (0.68, 4.44)		-	
≥ 1,8600	4.33 (1.29, 14.53)		-	
*Community contexts*				
Yangshan (rural)	Ref.	0.067		­
Zengcheng (suburban)	0.55 (0.28, 1.10)		-	
Tianhe (urban)	0.46 (0.23, 0.89)		-	
**Need factors**				
*Experienced influenza-like illnesses among family members in the past year*				
No	Ref.	0.282	-	-
Yes	1.83 (0.61, 5.53)		-	
*Adverse reactions to influenza vaccination of family members*				
No	Ref.	0.534	-	-
Yes	1.16 (0.31, 4.32)		-	
**Behavior factors**				
*Influenza vaccine history of children in the family*§				
No	Ref.	0.004	Ref.	0.037
Yes	2.97 (1.40, 2.67)		2.61 (1.06, 6.42)	
*Influenza vaccine history of older adults in the family*§				
No	Ref.	0.045	Ref.	0.039
Yes	3.27 (1.03, 10.36)		4.76(1.08, 20.90)	
**External environment factors**				
*Heard about negative information about influenza vaccine*				
No	Ref.	0.287	-	-
Yes	1.48 (0.72, 3.03)		-	
*Have friends/relatives object to the influenza vaccination*				
No	Ref.	0.534	-	-
Yes	0.85 (0.24, 2.97)		-	

* P value for univariate regression analysis

† Adjusted odds ratio: odds ratio adjusted for other covariates including participant type, sex, perceived vaccination effectiveness, trust in advice from healthcare personnel, perception of vaccination price and vaccination history of children and older people in the family in the multivariable logistic model

‡ P value for multivariable regression analysis

§ Variables entered multivariable logistic model

After the adjustment for confounders in the multivariable regression model (Table 3), participant type and previous utilization of influenza vaccination service in the family remained significantly associated with vaccine uptake. Older adults had a significantly lower likelihood of vaccine uptake compared to children (aOR = 0.19, 95%CI: 0.07–0.52, p = 0.001). Influenza vaccine history of children (aOR = 2.61, 1.06–6.42, p = 0.037) and older adults (aOR = 4.76, 1.08–20.90, p = 0.039) in the family were associated with a higher likelihood to vaccinate

### Univariable and multivariable regression analyses of the subsidized group

Univariable regression analysis results of the subsidized group (N = 300) are shown in Table 4. A higher vaccine uptake among children was also found compared to older people. Participants who were employed were more likely to take the vaccine compared to those unemployed or retired. Perceiving the vaccine to be safe, effective, trusting healthcare workers’ recommendations, and family members’ experience of influenza-like illnesses in the past year were associated with a higher likelihood to receive a vaccine

Multivariable regression analyses of the subsidized group (Table 4) also suggested a significantly lower vaccination rate among older people than that of children (aOR = 0.17, 0.08–0.36, p < 0.001). Participants who were married or lived with a partner (aOR = 0.32, 0.10–0.98, p = 0.046) had lower vaccination uptake than those who reported to be single. Recognition of vaccine effectiveness (aOR = 12.18, 5.21–28.50, p < 0.001), trusting recommendations from healthcare workers (aOR = 5.31, 1.79–15.80, p = 0.001), and having a family member who experienced influenza-like illnesses in the past year (aOR = 46.52, 4.10-533.78, p = 0.002) were associated with a higher likelihood of vaccination. Perceiving vaccine price as a barrier (aOR = 3.35, 1.32–8.41, p = 0.011) showed a positive correlation with influenza vaccine uptake

**Table 4:** Univariable analysis and multivariable analysis of associated factors for influenza vaccine uptake in the subsidized group in Guangdong Province, China, 2020–2021 (N = 300)

Explanatory variables	Crude OR (95% CI)	P-value*	Adjusted OR (95% CI) ^†^	P value‡
**Sociodemographic factors**				
*Participant type*§				
Children’s caregiver	Ref.	< 0.0001	Ref.	< 0.001
Older people	0.27 (0.15, 0.47)		0.17 (0.08, 0.36)	
*Age*	0.97 (0.95, 0.98)	< 0.0001	-	-
*Sex*§				
Male	Ref.	0.823	Ref.	0.066
Female	0.94 (0.52, 1.68)		0.47 (0.21, 1.05)	
*Completed highest education level*				
Primary school	Ref.	0.265	-	-
Middle school	1.05 (0.54, 2.04)		-	
Undergraduate or above	1.68 (0.80, 3.53)		-	
*Occupation*				
Unemployed/retired	Ref.	0.004	-	-
Farmer	2.29 (0.89, 5.87)		-	
Employed	2.73 (1.46, 5.10)		-	
*Marital status*§				
Single	Ref.	0.613	Ref.	0.046
Married or living with a partner	0.81 (0.35, 1.85)		0.32 (0.10, 0.98)	
**Belief factors**				
*Importance of influenza vaccine*				
Disagree	Ref.	0.719	-	-
Agree	0.88 (0.44, 1.75)		-	
*Safety of influenza vaccine*				
Disagree	Ref.	< 0.0001	-	-
Agree	4.44 (2.41, 8.17)		-	
*Effectiveness of influenza vaccine*§				
Disagree	Ref.	< 0.0001	Ref.	< 0.0001
Agree	7.36 (3.96,13.69)		12.18(5.21,28.50)	
*Trust the advice from medical personnel on influenza vaccine*§				
No	Ref.	< 0.0001	Ref.	0.001
Yes	7.88(3.89, 15.93)		4.95 (1.99, 12.43)	
*View the price of vaccines as barriers*§				
No	Ref.	0.330	Ref.	0.011
Yes	1.40 (0.71, 2.74)		3.35 (1.32, 8.41)	
**Enabling factors**				
*Annual income (USD)*				
< 1860	Ref.	0.711	-	-
1860 ~ 9300	0.91 (0.49, 1.67)		-	
9300 ~ 1,8600	1.04 (0.48, 2.25)		-	
≥ 1,8600	0.69 (0.53, 4.88)		-	
*Community resources*				
Yangshan (rural)	Ref.	0.067	-	-
Zengcheng (suburban)	0.55 (0.28, 1.10)		-	
Tianhe (urban)	0.46 (0.23, 0.89)		-	
**Need factors**				
*Experienced influenza-like illnesses among family members in the past year*§				
No	Ref.	0.002	Ref.	0.002
Yes	10.52(1.41,78.69)		46.52(4.10,533.78)	
*Adverse reactions to influenza vaccination of family members*				
No	Ref.	0.143	-	-
Yes	0.48 (0.16, 1.39)		-	
**Behavior factors**				
*Influenza vaccine history of children in the family*				
No	Ref.	0.272	-	-
Yes	1.36 (0.79, 2.36)		-	
*Influenza vaccine history of older adults in the family*				
No	Ref.	0.071	-	-
Yes	1.29 (0.94, 5.14)		-	
**External environment factors**				
*Heard about negative information about influenza vaccine*				
No	Ref.	0.374	-	-
Yes	0.77 (0.44, 1.36)		-	
*Have friends/relatives object to the influenza vaccination*				
No	Ref.	1.000	-	-
Yes	1.00 (0.35, 2.85)		-	

* P value for univariable analysis

† Adjusted odds ratio: odds ratio adjusted for other covariates including participant type, sex, perceived vaccination effectiveness, trust in advice from healthcare personnel, perception of vaccination price and vaccination history of children and older people in the family in the multivariable logistic model

‡ P value for multivariable analysis

§ Variables entered multivariable logistic model

## Discussion

There is limited understanding of barriers and facilitators of influenza vaccine uptake in LMICs [25, 26]. This study has added value by exploring the associated factors of influenza vaccine uptake among children and older adults based on different subsidized contexts in China. This analysis identified significantly lower influenza vaccine uptake among children and older adults in the self-paid group compared to those in the subsidized group, with older people less likely to be vaccinated than children in both groups. In the subsidized group, family members’ experience of influenza-like illnesses in the past year, positive attitudes towards vaccine effectiveness and suggestions from medical personnel were identified as associated factors of vaccine uptake. While in the self-paid group, influenza vaccination history in the family were associated with a higher likelihood to be vaccinated.

Participants in the self-paid group were less likely to be vaccinated than those in the subsidized group. This finding is supported by existing literature that reducing out-of-pocket expenses is an important facilitator for influenza vaccine uptake [12, 13, 37–39]. We also observed higher vaccination uptake in the self-paid group than previous observational studies [10, 40], and higher vaccination uptake in the subsidized group than places where free vaccination is provided [15]. These could be explained by three factors. First, use of educational materials such as the introductory pamphlet and medical personnel involvement in the study may have promoted awareness and adoption of vaccination behavior [41, 42]. Second, the COVID-19 pandemic may have increased awareness and acceptance of influenza vaccines given some shared symptoms and transmission routes between the two diseases [43]. Lastly, since study participants were recruited from clinics, it is possible that there is a self-selection bias and they may have a higher tendency of using vaccination services than those who use clinical services less frequently [16, 20, 22, 23, 28]

Our study also found that older people had significantly lower vaccine uptake than children. It is far below the World Health Assembly (WHA) target of 75% vaccine coverage among older people [44]. This may be partly attributable to who the key decision makers are for these two age groups. A Dutch survey [45] showed that parents deciding on behalf of their children were more positive about vaccination than adults who were making decisions for their own vaccination. This finding indicates that, in a rapidly ageing society in China, there is a pressing need for actions to improve the vaccine uptake among older people due to higher risks of severe illnesses or mortality once infected [46, 47]

In the subsidized vaccine group, positive perceptions and experience of influenza-like illness in the family played an important role in vaccine uptake. This is consistent with previous studies that showed positive impacts of participants’ confidence in vaccine effectiveness [11, 19, 20, 28] and healthcare workers’ suggestions [11, 19, 22]. These indicate that, in addition to lowering financial barriers by providing subsidized vaccines, health education and advocacy programs targeting improvement of public confidence in vaccines and healthcare providers may be important strategies to further enhance influenza vaccine uptake among high-risk groups. Existing literature [18] found lack of self-experience of influenza infection is a barrier to vaccination. Our findings suggest that experience of influenza-like illness among family members may be used to inform messages that can potentially improve people’s self-perceived susceptibility to influenza viruses and subsequent vaccination. These findings may be particularly relevant to places that are already offering free influenza vaccine services

In the self-paid group, previous vaccination experience was identified an associated factor with vaccine uptake. This is supported by previous studies [11, 19, 20]. Prior influenza vaccination experiences could shape positive attitudes towards the vaccine and contribute to subsequent vaccine uptake [48]. Since influenza viruses mutate frequently and annual vaccination is recommended [5], our findings demonstrate the importance of motivating people to receive their first influenza vaccination since this may contribute to the adoption of routine annual vaccination behavior and bring long-term benefits to the community

Our study findings have implications for tailoring strategies for influenza vaccine programs with varying levels of public funding support. In places where limited funds are provided and people self-pay for influenza vaccine services, messages and strategies targeting improved initiation of the influenza vaccination (e.g., subsidizing the first influenza vaccination for naive users) may be a promising direction. In regions where subsidized vaccines are available, in addition to motivating new users, additional efforts to improve public confidence in vaccine effectiveness and healthcare workers, and messages to change people’s self-perceived low risks of influenza infection are needed to enhance uptake. Additionally, compared to children, older adults are worth more attention, especially in an aging society

This study also has limitations. The data were collected at one given point in time without follow-ups and this has limitations to examine causality. First, future investigation can include longitudinal evaluation of influenza vaccine uptake in the next winter season, and our study findings provide important evidence base to support future tailored vaccine programs targeting improved uptake. Second, selection bias may result from the participant recruitment procedure. People recruited in attendance to primary care were more likely to have better health literacy. Additionally, lockdown restrictions after the COVID-19 outbreak have raised barriers to accessing primary healthcare, especially for those less-educated, deprived and unemployed groups [49]. Therefore, we anticipated that the participants in our study may be more amenable to accept vaccination compared to the general public. However, this study can still provide implications for practice of influenza vaccination promotion in the context of healthcare facilities (e.g., primary healthcare centers, etc.). To increase the study generalizability, future research to understand influenza vaccine uptake among a representative sample outside clinic settings stratified by funding contexts would be helpful. Future research to understand the adoption of influenza vaccines among a representative sample outside clinic settings may be helpful. Finally, measurements of perceived threats of the disease (e.g., perceived severity of influenza infection) and participants’ current health status (e.g., having a chronic illness or not) were not included in this study. But earlier studies have well documented their correlation with vaccine uptake [11, 16, 19, 20, 23, 29]

## Conclusions

Influenza vaccination disparity was found between older people and children in both subsidized and self-paid vaccine circumstances. Tailored interventions, particularly among older people, should be developed. Motivating new users to initiate influenza vaccination is an important step in context of self-paid vaccination. Addressing public confidence in vaccines/healthcare providers and people’s self-perceived low risks of influenza infection would be a promising direction for further improvement, and this may be particularly true for places that already provide free or subsidized influenza vaccines to their local populations

## Electronic supplementary material

Below is the link to the electronic supplementary material.

Supplementary Material 1

## Data Availability

The datasets used and/or analysed during the current study are available from the corresponding author on reasonable request.

## Abbreviations

aOR: Adjusted Odds Ratio
CI: Confidence interval
COVID-19: Coronavirus disease 2019
LMICs: Low and Middle Income Countries
OR: Odds Ratio
SD: Standard Deviation
UK: United Kingdom
US: United States
USD: United States Dollar
VIF: Variance Inflation Factor
WHA: World Health Assembly
