# Supplementary material for: Influenza vaccine uptake among children and older adults in China: a secondary analysis of a quasi-experimental study
Source: BMC Infect Dis. 2023 Apr 13;23:225. doi: 10.1186/s12879-023-08145-8 (PMC10098986; doi:10.1186/s12879-023-08145-8)
Supplement: Supplementary file 1 — Supplementary Material 1 [file 12879_2023_8145_MOESM1_ESM.docx]

**Appendix A1:**

**Appendix Fig A1. Pay-it-forward model overview**


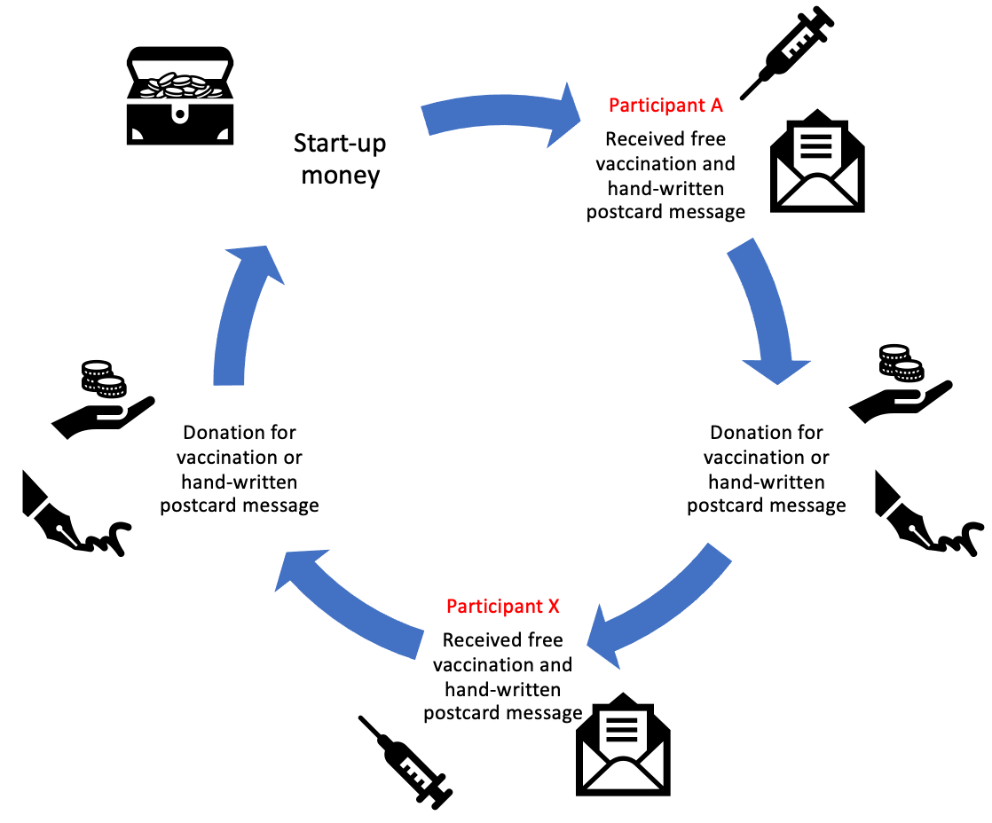


**Appendix A2: Detailed treatments of participants in the three original arm:**

1) Standard of care arm (i.e., control group): Participants in this study arm were exposed to the typical standard of care for that clinic. They were provided with a brief introduction to the influenza vaccine using a pamphlet (**Appendix B2**) and then asked if they were willing to pay out of pocket at the standard market price patient prices CN¥55-153) of an influenza vaccine.

2) Pay-it-forward arm: participants recruited in pay-it-forward group where the same introductory pamphlet was provided. Project staff also explained the pay-it-forward program with the PIF pamphlet (**Appendix B3**), including the purpose, the opportunity to receive one dose of influenza vaccination for free, and the decisions to vaccinate and donate money. Participants were told that the patient prices of an influenza vaccine including administration fees for children were CN¥65 and for adults ranged from CN¥82-153 respectively (the vaccines used in different sites are different, affected by the local availability), while former families had donated money to cover the costs and also created handwritten postcards. If they decided to accept a vaccination, they were then asked whether they were willing to donate any amount of money to support more future participants to receive the same vaccination service, they were also invited to write anonymous postcard messages for future users. It is assured that the donation was completely voluntary, and any donation amount was acceptable.

3) Free-of-charge arm: participants in the free-of-charge condition were invited using the same pamphlet and were provided with free influenza vaccination, but they did not receive any community created messages about the pay-it-forward program.

**Appendix A3: Pilot study and sample size**

A pilot study was done at the rural study site from December 2019 to April 29, 2020. In the pilot feasibility trial, 91% (41/44) participants in the pay-it-forward group and 23% (13/57) of participants in the standard-of-care group received an influenza vaccination. We estimated 30% of vaccine uptake rate in the standard-of-care arm and 80% in the pay-it-forward arm based on our pilot data. Sample size calculations were stratified by age groups due to the differences in sociodemographic determinants of influenza vaccine uptake between children and older adults. Given 90% power to test that the pay-it-forward intervention is superior to the standard-of-care condition in promoting vaccination uptake, a sample size of 100 participants (50 participants in the control group and 50 in the intervention group) for each age group should be obtained give, with a margin of 10% and a significance level of 0.025. Sample size was increased by 50% to allow for the secondary analyses, resulting in a sample size of 75 participants for each age group in each group. A free-of-charge arm (n=150, 75 children and 75 older adults), in which participants were offered free influenza vaccination was included to compare the cost of pay-it-forward interventions with free-vaccination provision. Thus, a total sample of 450 participants (150 children and 150 older people) was targeted.

# **Appendix B: Supplementary marterials used in primary study**

### **Appendix B1: Online questionnaire example**

**
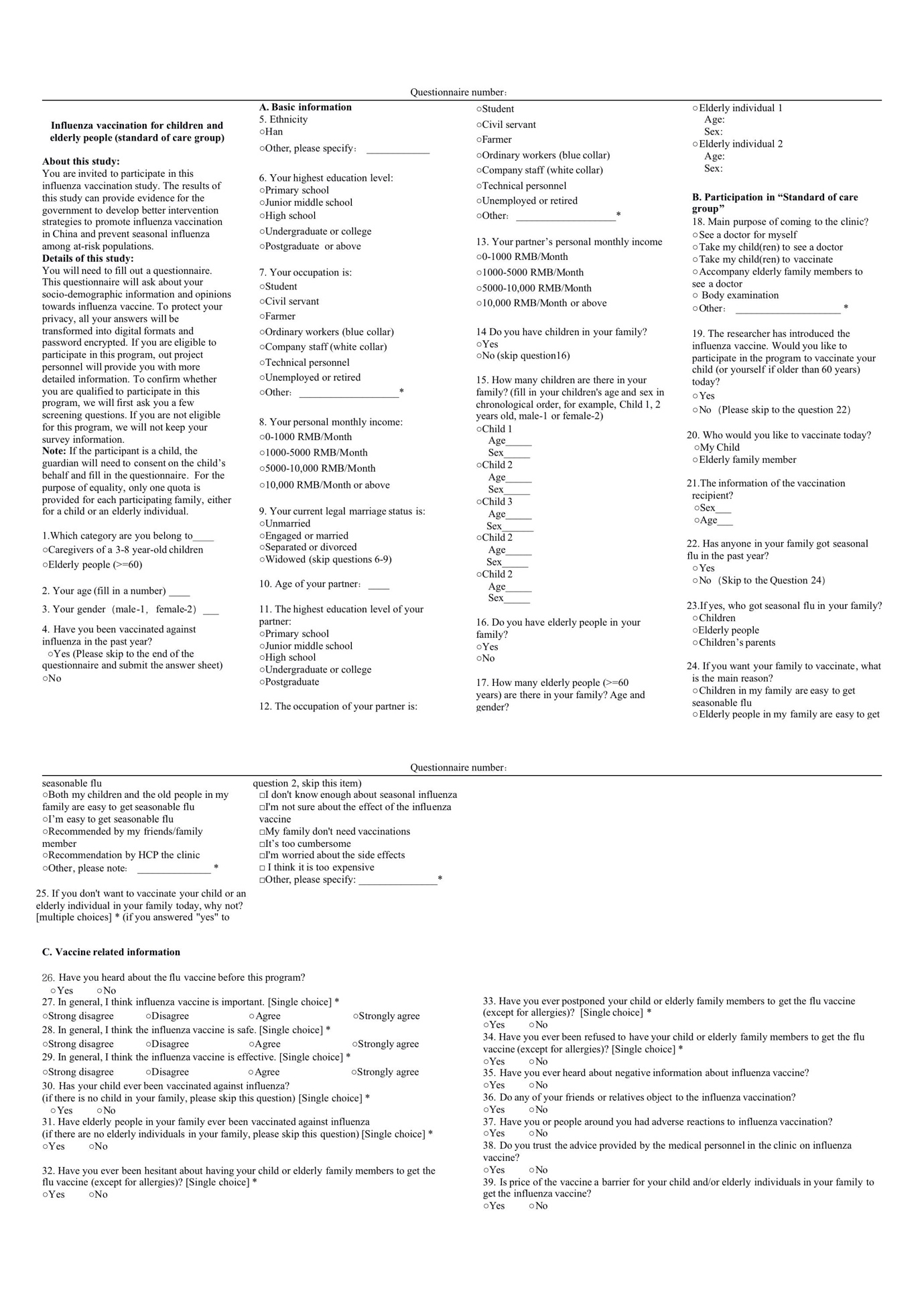
**

### **Appendix B2: Influenza and influenza vaccine introduction pamphlet**

### **in English**


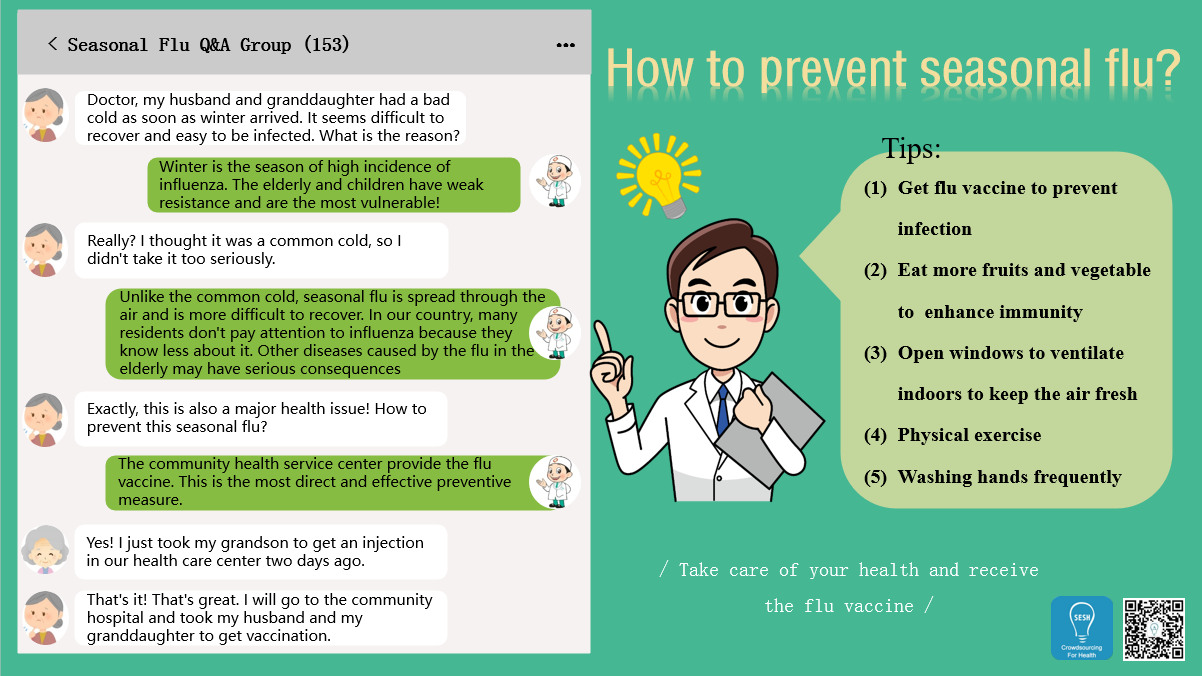


### **Appendix B3: Pay-it-forward project introduction pamphlet in English**


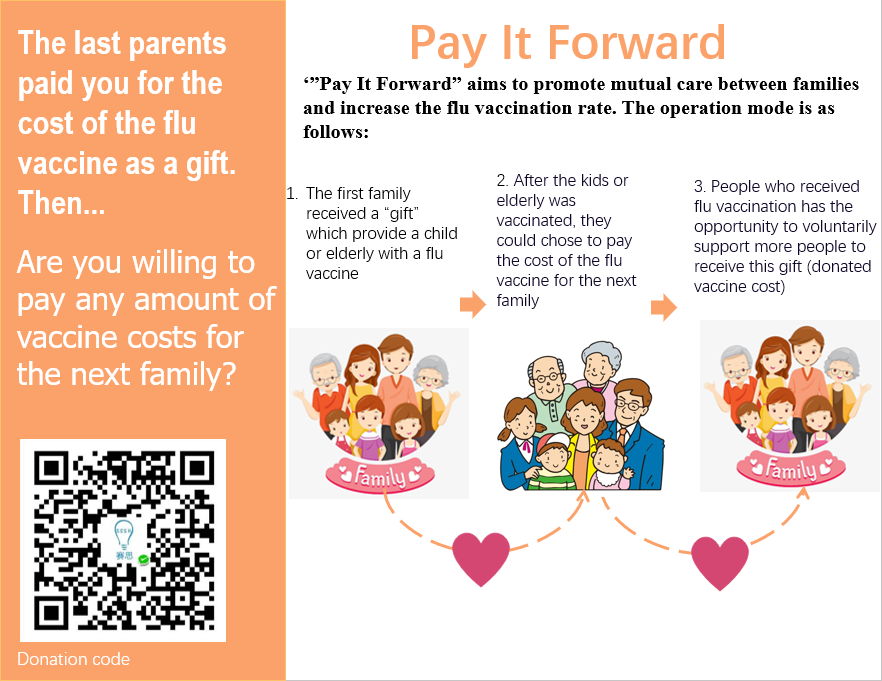


**Appendix C: Supplementary results**

**Figure C1. Influenza vaccine uptake rates in self-paid and subsidized groups by participant types in Guangdong Province, China, 2020-2021 (N=450)**

**Appendix Table C2. Missing value of different intervention arms in Guangdong Province, China, 2020-2021 (N=450)**

| **Characteristics** | **No. of missing values* (%)** | | | |
| --- | --- | --- | --- | --- |
|  | **Total**  **(N=450)** | **Standard-of-care group**  **(N=150)** | **Pay-it-forward**  **group**  **(N=150)** | **Free-of-charge**  **group**  **(N=150)** |
| **Sociodemographic factors** |  |  |  |  |
| **Participant type** | 0 | 0 | 0 | 0 |
| **Age (years)** | 0 | 0 | 0 | 0 |
| **Sex** | 0 | 0 | 0 | 0 |
| **Ethnicity** | 0 | 0 | 0 | 0 |
| **Completed highest education level** | 0 | 0 | 0 | 0 |
| **Occupation** | 0 | 0 | 0 | 0 |
| **Marital status** | 0 | 0 | 0 | 0 |
| **Belief factors** |  |  |  |  |
| **I think influenza vaccine is important** | 15 (3.0%) | 2 (1.3%) | 6 (4.0%) | 7 (4.7%) |
| **I think the influenza vaccine is safe** | 15 (3.0%) | 9 (6.0%) | 6 (4.0%) | 0 (0.0%) |
| **I think the influenza vaccine is effective** | 15 (3.0%) | 9 (6.0%) | 6 (4.0%) | 0 (0.0%) |
| **Trust the advice from medical personnel on influenza vaccine** | 0 | 0 | 0 | 0 |
| **View the price of vaccines as barriers** | 0 | 0 | 0 | 0 |
| **Enabling factors** |  |  |  |  |
| **Annual income**  **(USD)** | 0 | 0 | 0 | 0 |
| **Community resources** | 0 | 0 | 0 | 0 |
| **Needs factors** |  |  |  |  |
| **Experienced influenza-like illnesses among family members in the past year** | 0 | 0 | 0 | 0 |
| **Adverse reactions to influenza vaccination of family members** | 0 | 0 | 0 | 0 |
| **Behaviour factors** |  |  |  |  |
| **Influenza vaccine history of children in the family** | 45 (10.0%) | 27 (18.0%) | 9 (6.0%) | 9 (6.0%) |
| **Influenza vaccine history of older adults in the family** | 30 (6.7%) | 15 (10.0%) | 4 (2.7%) | 11 (7.3%) |
| **External environmental factors** |  |  |  |  |
| **Have ever heard about negative information about influenza vaccine** | 0 | 0 | 0 | 0 |
| **Have friends/relatives object to the influenza vaccination** | 0 | 0 | 0 | 0 |

* Missing values indicates the missing data caused by the participants who did not answer the question

###

### **Appendix Table C3. Descriptive analysis of sociodemographic characteristics**

**within the subsidized arm in Guangdong Province,**

**China, 2020-2021 (N=300)**

| **Sociodemographic factors** | **Total of subsidized group**  **(N=300)**  **(%)** | **Subsidized group** | | **P value*** |
| --- | --- | --- | --- | --- |
|  |  | **Pay-it-forward**  **arm**  **(N=150)** | **Free-of-charge**  **arm**  **(N=150)** |  |
|  |  |  |  |  |
| **Participant type** |  |  |  |  |
| Children’s caregiver | 150 (50.0%) | 75 (50.0%) | 75 (50.0%) | 1.000 |
| Older adults | 150 (50.0%) | 75 (50.0%) | 75 (50.0%) |  |
| **Age, Mean (SD)** | 52 (17.7) | 52 (17.1) | 53 (17.6) | 0.717 |
| **Sex** |  |  |  |  |
| Male | 83 (27.7%) | 43 (28.7%) | 40 (26.7%) | 0.699 |
| Female | 217 (72.3%) | 107 (71.3%) | 110 (73.3%) |  |
| **Completed highest education level** |  |  |  |  |
| Primary school | 63 (21.0%) | 30 (20.0%) | 33 (22.0%) | 0.809 |
| Middle school | 138 (46.0%) | 68 (45.3%) | 70 (46.7%) |  |
| Undergraduate or above | 132 (29.3%) | 52 (34.7%) | 47 (31.3%) |  |
| **Occupation** |  |  |  |  |
| Unemployed/retired | 234 (52.0%) | 79 (52.7%) | 82 (54.7%) | 0.814 |
| Peasant | 54 (12.0%) | 16 (10.7%) | 18 (12.0%) |  |
| Employed | 99 (33.0%) | 55 (36.7%) | 50 (33.3%) |  |
| **Marital status** |  |  |  |  |
| Single | 37 (12.3%) | 24 (16.0%) | 13 (8.7%) | 0.053 |
| Married or living with a partner | 263 (87.7%) | 126 (84.0%) | 137 (91.3%) |  |

*P-value from chi-square test for comparison of proportion and t test for comparison of mean.

**Appendix Table C4. Univariate analysis of associated factors for influenza vaccine uptake in pay-it-forward and free-of-charge arm in Guangdong Province,**

**China, 2020-2021 (N=300)**

|  | **Pay-it-forward arm (N=150)** | | **Free-of-charge arm (N=150)** | | **P-value of**  **Heterogeneity of OR**† |
| --- | --- | --- | --- | --- | --- |
| **Explanatory variables** | **Crude OR**  **(95% CI)** | **P-value of LRT*** | **Crude OR (95% CI)** | **P-value of LRT*** |  |
|  |  |  |  |  |  |
| **Sociodemographic factors** |  |  |  |  |  |
| **Participant type** |  |  |  |  |  |
| Children’s care giver | Ref. | <0.0001 | Ref. | 0.009 | 0.398 |
| Older adults | 0.20 (0.09,0.47) |  | 0.34 (0.15, 0.76) |  |  |
| **Age** | 0.97 (0.94, 0.99) | 0.003 | 0.96 (0.94, 0.98) | <0.0001 | 0.908 |
| **Sex** |  |  |  |  |  |
| male | Ref. | 0.371 | Ref. | 0.600 | 0.288 |
| female | 0.68 (0.29, 1.59) |  | 1.29 (0.57, 2.94) |  |  |
| **Education** |  |  |  |  |  |
| primary school | Ref. | 0.007 | Ref. | 0.106 | 0.834 |
| middle school | 0.40 (0.15, 1.12) |  | 2.76 (1.08, 7.09) |  |  |
| Undergraduate/above | 1.61 (0.49, 5.32) |  | 6.67 (0.63, 4.38) |  |  |
| **Occupation** |  |  |  |  |  |
| Unemployed/retired | Ref. | 0.037 | Ref. | 0.052  (Fisher) | 0.843 |
| peasant | 1.56 (0.46, 5.29) |  | 3.71(0.79,17.36) |  |  |
| employed | 3.05 (1.26, 7.37) |  | 2.43 (1.00, 5.92) |  |  |
| **Marital status** |  |  |  |  |  |
| Single | Ref. | 0.190 | Ref. | 0.381 | 0.226 |
| Married or living with a partner | 0.52 (0.17, 1.63) |  | 1.46 (0.42, 5.05) |  |  |
| **Belief factors** |  |  |  |  |  |
| **I think influenza vaccine is important** |  |  |  |  |  |
| Disagree | Ref. | 0.969 | Ref. | 0.548 | 0.632 |
| Agree | 1.02 (0.41, 2.53) |  | 0.72 (0.25, 2.09) |  |  |
| **I think the influenza vaccine is safe** |  |  |  |  |  |
| Disagree | Ref. | <0.0001 | Ref. | 0.031 | 0.108 |
| Agree | 8.33(3.20, 21.67) |  | 2.82 (1.24, 6.40) |  |  |
| **I think the influenza vaccine is effective** |  |  |  |  |  |
| Disagree | Ref. | <0.0001 | Ref. | <0.0001 | 0.446 |
| Agree | 10.92(3.95,30.22) |  | 6.26(2.75,14.21) |  |  |
| **Trust the advice from medical personnel on influenza vaccine** |  |  |  |  |  |
| No | Ref. | <0.0001 | Ref. | 0.001 | 0.270 |
| Yes | 12.17(4.30,34.48) |  | 5.13(1.92,13.72) |  |  |
| **View the price of vaccines as barriers** |  |  |  |  |  |
| No | Ref. | 0.978 | Ref. | 0.184 | 0.325 |
| Yes | 1.01 (0.41, 2.50) |  | 2.02 (0.72, 5.69) |  |  |
| **Enabling factors** |  |  |  |  |  |
| **Annual income**  **(USD)** |  |  |  |  |  |
| <1860 | Ref. | 0.079 | Ref. | 0.781 | 0.649 |
| 1860~9300 | 0.66 (0.29, 1.55) |  | 1.29 (0.53, 3.12) |  |  |
| 9300~1,8600 | 0.92 (0.24, 1.99) |  | 1.63 (0.51, 5.22) |  |  |
| ≥1,8600 | - |  | 0.89 (0.26, 3.03) |  |  |
| **Community resources** |  |  |  |  |  |
| Yangshan (rural) | Ref. | 0.003 | Ref. | 0.021 | 0.058 |
| Zengcheng (suburban) | 1.15 (0.41, 3.18) |  | 0.31 (0.12, 0.80) |  |  |
| Tianhe (urban) | 0.28 (0.11, 0.70) |  | 0.86 (0.31, 2.46) |  |  |
| **Needs factors** |  |  |  |  |  |
| **Experienced influenza-like illnesses among family members in the past year** |  |  |  |  |  |
| No | Ref. | 0.416 | Ref. | § | 0.081 |
| Yes | 2.17 (0.25,18.63) |  | § |  |  |
| **Adverse reactions to influenza vaccination of family members** |  |  |  |  |  |
| No | Ref. | 0.111 | Ref. | 0.446 | 0.311 |
| Yes | 0.22 (0.04, 1.37) |  | 0.72 (0.18, 2.94) |  |  |
| **Behavior factors** |  |  |  |  |  |
| **Influenza vaccine history of children in the family** |  |  |  |  |  |
| No | Ref. | 0.059 | Ref. | 0.729 | 0.111 |
| Yes | 2.15 (0.97, 4.78) |  | 0.87 (0.40, 1.89) |  |  |
| **Influenza vaccine history of older adults in the family** |  |  |  |  |  |
| No | Ref. | 0.081 | Ref. | 0.236 | 0.988 |
| Yes | 2.30 (0.82, 6.46) |  | 2.26(0.48,10.57) |  |  |
| **External context factors** |  |  |  |  |  |
| **Have ever heard about negative information about influenza vaccine** |  |  |  |  |  |
| No | Ref. | 0.887 | Ref. | 0.259 | 0.493 |
| Yes | 0.94 (0.42, 2.13) |  | 0.63 (0.28, 1.40) |  |  |
| **Have friends/relatives object to the influenza vaccination** |  |  |  |  |  |
| No | Ref. | 0.213 | Ref. | 0.195 | 0.088 |
| Yes | 3.35 (0.41, 27.36) |  | 0.44 (0.12, 1.67) |  |  |

*P-value of Likelihood ratio test in logistic regression

† P-value of odds ratio heterogeneity in Mantel-Haenszel analysis

§Values removed by logistic model in Stata for too few observations in subgroups

**Appendix Table C5. Collinearity diagnosis of self-paid group in Guangdong Province,**

**China, 2020-2021 (N=150)**

| **Explanatory variables*** | **VIF** | **SQRT**  **VIF** | **Tolerance** | **R-Squared** |
| --- | --- | --- | --- | --- |
| **Sociodemographic factors** |  |  |  |  |
| Age | 5.83 | 2.41 | 0.17 | 0.83 |
| Participants type | 5.64 | 2.37 | 0.18 | 0.82 |
| Sex | 1.11 | 1.05 | 0.90 | 0.10 |
| **Belief factors** |  |  |  |  |
| I think the influenza vaccine is safe | 1.26 | 1.12 | 0.79 | 0.21 |
| I think the influenza vaccine is effective | 1.41 | 1.19 | 0.71 | 0.29 |
| View the price of vaccines as barriers | 1.18 | 1.09 | 0.85 | 0.15 |
| Trust the advice from medical personnel on influenza vaccine | 1.24 | 1.11 | 0.81 | 0.19 |
| **Behaviour factors** |  |  |  |  |
| Influenza vaccine history of children in the family | 1.18 | 1.08 | 0.85 | 0.15 |
| Influenza vaccine history of older adults in the family | 1.11 | 1.06 | 0.90 | 0.10 |

*Explanatory variables selected for collinearity diagnosis were those variables with significant criteria<0.2 after the adjustment of prior confounder

**Appendix Table C6. Collinearity diagnosis of subsidized group in Guangdong Province,**

**China, 2020-2021 (N=300)**

| **Explanatory variables*** | **VIF** | **SQRT**  **VIF** | **Tolerance** | **R-Squared** |
| --- | --- | --- | --- | --- |
| **Sociodemographic factors** |  |  |  |  |
| Age | 6.63 | 2.57 | 0.15 | 0.84 |
| Participants type | 6.05 | 2.46 | 0.17 | 0.83 |
| Sex | 1.11 | 1.05 | 0.90 | 0.10 |
| Occupation | 1.85 | 1.36 | 0.54 | 0.46 |
| Marital status | 1.19 | 1.09 | 0.84 | 0.16 |
| **Belief factors** |  |  |  |  |
| I think the influenza vaccine is safe | 1.35 | 1.16 | 0.74 | 0.26 |
| I think the influenza vaccine is effective | 1.47 | 1.21 | 0.68 | 0.32 |
| Trust the advice from medical personnel on influenza vaccine | 1.14 | 1.07 | 0.88 | 0.12 |
| View the price of vaccines as barriers | 1.12 | 1.06 | 0.89 | 0.11 |
| **Enabling factors** |  |  |  |  |
| Community resources | 1.11 | 1.06 | 0.90 | 0.10 |
| **Needs factors** |  |  |  |  |
| Experienced influenza-like illnesses among family members in the past year | 1.03 | 1.02 | 0.97 | 0.03 |
| Adverse reactions to influenza vaccination of family members | 1.04 | 1.02 | 0.96 | 0.04 |
| **Behaviour factors** |  |  |  |  |
| Influenza vaccine history of children in the family | 1.10 | 1.05 | 0.91 | 0.09 |
| Influenza vaccine history of older adults in the family | 1.13 | 1.06 | 0.89 | 0.11 |

*Explanatory variables selected for collinearity diagnosis were those variables with significant criteria<0.2 after the adjustment of prior confounder
